# Open-label: the clinical effects of adding cannbidiol to usual care of patients with residual symptoms in the diagnosis of Obsessive Compulsive Disorder

**DOI:** 10.1192/j.eurpsy.2023.524

**Published:** 2023-07-19

**Authors:** E. R. De Morais, A. W. Z. P. W. Zuardi, F. S. Guimarães, F. L. Osório, J. E. Hallak, R. F. Sanches, J. A. S. Crippa

**Affiliations:** ^1^Neuroscience and Behavior Department, Faculty of Medicine of Ribeirão Preto University of São Paulo, Ribeirão Preto, Brazil

## Abstract

**Introduction:**

Obsessive-compulsive disorder (OCD) is a heterogeneous and debilitating neuropsychiatric disorder. First-line antidepressants with Selective Serotonin Reuptake Inhibitors (SSRIs) and Clomipramine (a tricyclic antidepressant) are unresponsive or partially responsive in 40% of treated patients. Preclinical studies have shown that cannabidiol (CBD) can reduce compulsive behavior in animals, and considering that the release of glutamate in the action of CBD can inhibit terminal axons of neurons in the corticostriatal-thalamo-cortical circuit, we chosefor testing CBD, a drug with few side effects and low toxicity, as an adjuvant in treating OCD.

**Objectives:**

To evaluate the clinical effects of CBD add-on to the usual pharmacological treatment of outpatients diagnosed with OCD.

**Methods:**

Methods: This is an open-label study in which patients received CBD 300mg-day for 30 days in addition to their usual treatments and CBD 600mg-day for an additional 30 days if they have not reduced at least 25% of symptoms compared to the baseline evaluated by the Yale-brown obsessive-compulsive scale (Y-BOCS). Psychometric scales were used to assess the effects of CBD: Y-BOCS, General Anxiety Disorder 7 (GAD-7), Clinical Overall Impressions-Severity (CGI-S), Clinical Global Impressions-Improvement (CGI-I), Patient Health Questionnaire-9 (PHQ-9), Epworth Sleepiness Scale and Udvalg Scale for Kliniske Undersogelser (UKU) scale.

Participants: We initially assessed 33 participants in the study. So far, 19 patients have completed the study. Eleven did not complete the study, and 03 patients are still in the study trial.

**Results:**

Mean (SD) Y-BOCS scores decreased from 28.0 (4.8) at baseline to 13.8 (8.6) at week 8, corresponding to a - 50.7% reduction (P<.0001). Anxiety symptoms (GAD-7 scale) (P=0.001) and CGI-Severity scales scores (P<0.001) improved significantly.

**Table:** 

Measure	Baseline, Mean (SD)	Week 4, Mean (SD)	Week 8, Mean (SD)	Mean Change (Baseline to Week 4)	Mean Change (Baseline to Week 8)				
				Mean (SD)		p-value		t-test	
Y-Bocks	28.0 (4.8)	17.8 (7.3)	13.8 (8.6)	10.1 (6.5)	<0,001	7.1	14.1 (7.9)	<0,001	7.7
GAD-7	13.8 (4.4)	8.14 (4.9)	8.0 (5.7)	5.7 (6.4)	0,001	4.0	5.6 (5.8)	0,001	4.1
CGI-I	5.48 (0.9)	3.9 (1.3)	3.3 (1.5)	1.5 (1.3)	<0,001	5.28	2.1 (1.2)	<0,001	7.4

a n=22;

b n=19

**Image:**

**Figure:**
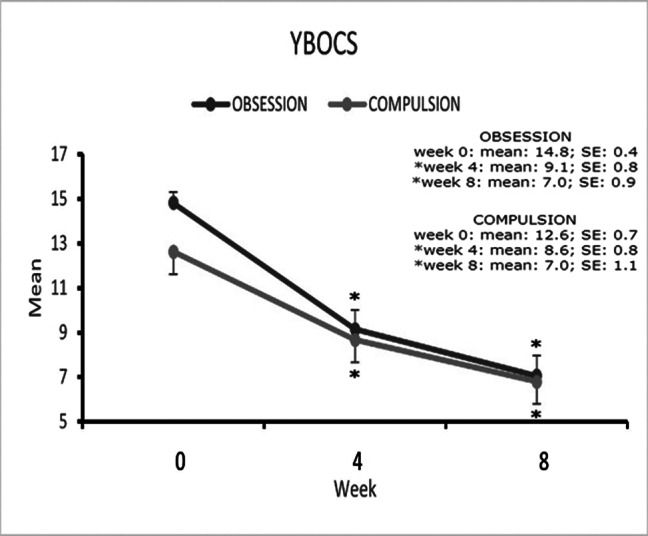

**Image 2:**

**Figure:**
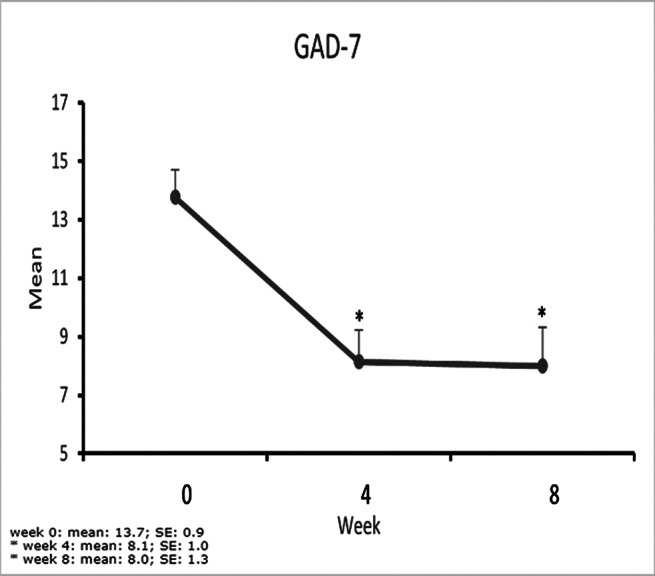

**Image 3:**

**Figure:**
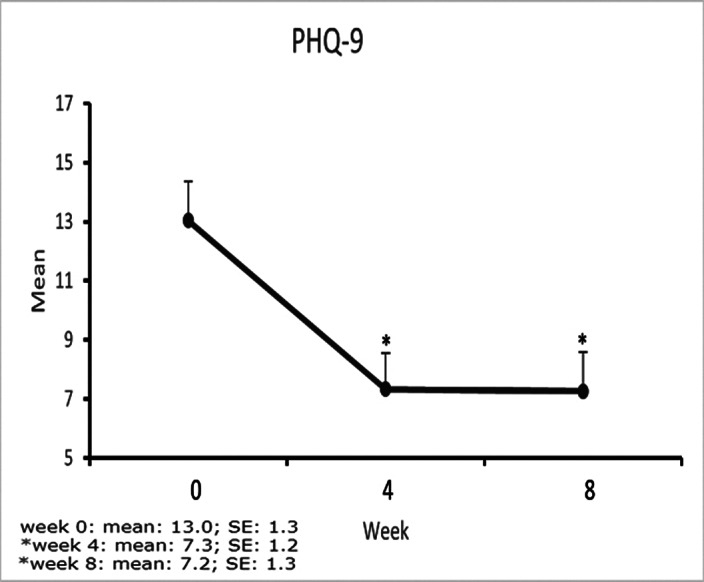

**Conclusions:**

We concluded that there was an important reduction in the psychometric scales used with statistical significance. This is a partial analysis of the results as the study progresses. We believe that in the future we will be able to complete this study and have a better analysis of the results that are promising. At the end of the study, other psychometric scales such as CGI-I, epworth scale and UKU will be analyzed.

**Disclosure of Interest:**

None Declared

